# Intraoperative Periprosthetic Proximal Femoral Fractures During Direct Anterior Approach: A New Screw and Plate Fixation Method

**DOI:** 10.3390/jcm15124765

**Published:** 2026-06-19

**Authors:** Filippo Randelli, Francesco Manzini, Alberto Fioruzzi, Jacopo Menini, Giuseppe Fedele, Clemente Caria

**Affiliations:** 1 Hip Department (CAD), ASST Gaetano Pini-CTO, University of Milan, 20122 Milano, Italy; 2 Istituti Clinici Zucchi-Monza-Gruppo San Donato, 20900 Monza, Italy; 3 Department of Orthopaedics and Trauma Surgery, ASST Gaetano Pini-CTO, University of Milan, 20122 Milano, Italy; 4 Department of Orthopaedics and Trauma Surgery, University Campus Bio-Medico, 00128 Rome, Italy

**Keywords:** periprosthetic femoral fracture, direct anterior approach, total hip arthroplasty, lag screw fixation, locking plate fixation

## Abstract

**Background:** Intraoperative periprosthetic proximal femoral fractures (PPFFs) represent a significant complication during total hip arthroplasty (THA), especially when using cementless stems via a direct anterior approach (DAA). **Methods:** This retrospective case series evaluated 10 patients with Vancouver A2 PPFFs treated with 2.7 mm lag screws alone or in combination with plates during DAA THA or partial hip arthroplasty between January 2021 and March 2024. **Results:** All fractures healed. One patient experienced 1 cm of stem subsidence without the need for revision. The mean Harris Hip Score improved from 35.4 preoperatively to 85.6 postoperatively. Functional recovery and radiological stability were comparable between fixation methods, though the screw-only group experienced slightly more postoperative pain. Patients in the screw-and-plate group were significantly older than those in the screw-only group (*p* = 0.026). No significant differences were found between groups regarding surgical time (*p* = 0.62) or BMI (*p* = 0.82). Due to the limited number of subsidence events, the statistical comparison of subsidence rates was inconclusive. **Conclusions:** In this preliminary retrospective case series, the use of 2.7 mm lag screws and small locking plates appeared feasible and was associated with favorable short-term outcomes in selected Vancouver A2 intraoperative PPFFs during DAA. These findings are hypothesis-generating and require confirmation in larger, prospective comparative studies.

## 1. Introduction

Intraoperative periprosthetic proximal femoral fractures (PPFFs) represent a significant complication during primary total hip arthroplasty (THA), particularly with cementless stems through a direct anterior approach (DAA), with an incidence that varies from 2% to more than 10% [1,2,3,4,5,6]. These fractures can compromise implant stability and lead to poor outcomes, particularly if not adequately managed. Both patient- and surgery-related factors contribute to their occurrence [7]. Patient factors associated with replacement include female sex, age < 60 or >80, hip dysplasia, varus neck, narrow proximal canal, Dorr type C femurs, elevated body mass index, osteoporosis, and femoral neck fracture [2,4,8,9]. Surgical factors include the use of cementless implants, implant designs, and surgeon experience [10].

While simple calcar fractures can often be managed with a single compression screw, more complex A2 patterns present a distinct and less studied clinical challenge.

The proximal femur plays a pivotal role in ensuring implant stability and functionality through tendon attachments at the metaphyseal level. While the calcar has been extensively studied, other areas of the proximal femur remain less explored, despite their importance [11,12]. According to the Vancouver intraoperative periprosthetic fracture classification, the cases in this series correspond to Type A2 fractures, which are proximal metaphyseal fractures involving the calcar region, sometimes extending beyond the lesser trochanter [13,14].

In recent years, the direct anterior approach (DAA) has gained popularity due to its minimally invasive nature and faster recovery [15]. However, this approach is associated with a higher risk of intraoperative PPFF. Moreover, PPFFs during a DAA are challenging to manage due to limited exposure. Fractures at the base of the greater trochanter (GT) or the proximal anterior or posterior cortex are the most complex to fix through a DAA. Traditionally, cerclage wires have been used to treat intraoperative proximal femoral fractures, sparing the greater trochanter. While effective in preventing fracture propagation, cerclage fixation requires extensive soft tissue dissection and is less effective against shear and rotational forces [16,17]. Despite the recognized risk of intraoperative periprosthetic proximal femoral fractures (PPFFs) during DAA, there is a knowledge gap regarding minimally invasive alternatives that preserve soft tissue and provide stable fixation, particularly in cases of more complex Vancouver A2-type fractures. An interesting biomechanical study has suggested that single compression screws, placed perpendicular to the fracture line, may offer fixation strength comparable to that of cerclage wires in calcar fractures [18]. This technique has the advantage of requiring less dissection, making it particularly suitable for managing fractures during DAA. Unfortunately, more complex PPFFs, rather than simple calcar fractures, cannot be treated successfully with only a compression screw.

Over the last few years, we have employed a new method to treat Vancouver A2 periprosthetic proximal femoral fractures that occur during DAA in our department. We have fixed various Vancouver A2 PPFFs using 2.7 mm lag screws alone or in combination with plates, depending on the fracture location, pattern, and length [14].

Therefore, this study aimed to describe and evaluate a minimally invasive fixation technique using 2.7 mm lag screws, with or without small locking plates, for intraoperative Vancouver A2 periprosthetic proximal femoral fractures during DAA THA and PHA.

## 2. Materials and Methods

This study reviewed patients who experienced A2 periprosthetic intraoperative proximal femoral fractures during total hip arthroplasty (THA) or partial hip arthroplasty (PHA) via DAA between January 2021 and March 2024. The study was conducted in accordance with the Declaration of Helsinki and was approved by the Institutional Review Board of our hospital (protocol CTS-P-2026-04). Among 1105 consecutive DAA procedures (THA and PHA) performed at our institution between January 2021 and March 2024, intraoperative Vancouver A2 periprosthetic proximal femoral fractures were identified in 10 cases. All 10 met the inclusion criteria and were included in the analysis. The fixation method employed off-label use of the DePuy Synthes Variable Angle LCP™ Forefoot–Midfoot System (DePuy Synthes, Warsaw, IN, USA) on the proximal femur; this use was included in the approved protocol. Written informed consent for study participation and implantation of the device was obtained from all patients before surgery.

### 2.1. Study Population and Criteria

Inclusion criteria consisted of patients sustaining intraoperative fractures involving the calcar region or adjacent anterior or posterior cortex up to the base of the greater trochanter, managed using 2.7 mm lag screws (DePuy Synthes Cortex Screws; DePuy Synthes, Warsaw, IN, USA) either alone or in conjunction with plates (DePuy Synthes Straight and L-Fusion Plates 2.4/2.7 mm, lengths ranging from 37 to 62 mm; DePuy Synthes, Warsaw, IN, USA). Exclusion criteria included incomplete documentation, follow-up duration less than six months, or fractures treated with alternative fixation methods. Demographic data, including age, sex, BMI, comorbidities, and fracture characteristics, were collected from medical records. For the purpose of this study, “simple” fractures were defined as non-comminuted calcar fractures without distal propagation, whereas “complex” fractures included multifragmentary patterns and/or posterior, anterior, lateral, or distal extension (Table 1).

### 2.2. Radiographic and Clinical Evaluation

Radiographic evaluations were conducted at multiple intervals: within 48 h postoperatively and at 1, 2, and 6 months, followed by annual assessments. Key radiographic outcomes were evaluated:

- Fracture healing, nonunion, or malunion.
- Stem migration, measured as the distance from the greater trochanter to the femoral stem’s shoulder using calibrated digital radiographs [19].
- Signs of implant loosening or hardware failure [20].

Clinical outcomes were assessed using the Harris Hip Score (HHS) to measure functional recovery, pain levels, and mobility. Complications were also considered.

Descriptive statistics were utilized to summarize patient demographics, surgical outcomes, and follow-up findings. Categorical variables were presented as frequencies and percentages, while continuous variables were expressed as means and standard deviations. Where applicable, outcomes were compared between patients treated with screws alone and those receiving plates and screws, focusing on radiological stability, fracture healing, and functional recovery metrics [13]. The primary endpoint was fracture healing at the final follow-up, defined radiographically as bridging callus on at least two cortices. Secondary endpoints included stem migration, signs of implant loosening or hardware failure, the Harris Hip Score, and the occurrence of complications.

### 2.3. Surgical Technique and Hardware

All patients underwent surgery through a DAA without the use of a traction table, with the patient in a supine position. As preparation for a safe femoral stem insertion, a meticulous posterior capsule release was always performed, including, if needed, the detachment of piriformis and triceps coxae tendons (superior gemellus, obturator internus, and inferior gemellus) from the posterior GT. However, the obturator externus and quadratus femoris muscles were always preserved. This approach enables excellent elevation and visualization of the proximal femur, minimizing intraoperative complications.

During femoral canal broaching and after insertion of the definitive stem, a meticulous inspection of the proximal femur was conducted before proceeding with the trial or final reduction.

In the case of a visible fracture line on the proximal femur, a meticulous soft tissue elevation over a few centimeters was performed to assess possible fracture propagation and plan the fixation. When the fracture extended from the medial to the anterior cortex, a limited elevation of the vastus lateralis muscle was necessary to assess and manage the fracture properly. In contrast, when the fracture line extended posteriorly and laterally from the medial side, a broader dissection of the external rotator muscles became essential. In some instances where the fracture was centered on the calcar and extended distally, more extensive dissection and exposure of the medial joint capsule were required, occasionally involving the partial release of the psoas tendon to achieve sufficient exposure and facilitate fixation.

Key factors guiding decision-making in the event of an intraoperative fracture included bone quality, fracture location (calcar, posterior/posterolateral, and anterior/anterolateral cortex), fracture direction, and distal extension. These parameters determined whether a single screw would be sufficient or whether additional screws or a plate were required for stable fixation.

Fixation choice followed a structured decision algorithm:

**Screws only:** short, well-defined calcar fractures with good bone quality, without distal propagation.

**Additional screws:** added when the fracture extension allowed multiple points of interfragmentary fixation.

**Screws plus plate:** fractures with posterior, anterior, lateral, or anterolateral extension, poor bone quality, or a perceived risk of greater trochanter detachment. Screws were typically placed perpendicular to the fracture line to provide interfragmentary compression, and plates were positioned along the lateral or anterolateral cortex according to the fracture’s orientation. This structured pathway reflects the intraoperative decision-making process used in our series. It has not been validated as a formal protocol and should be interpreted as a description of our institutional practice.

The surgical hardware employed for managing the intraoperative fracture consisted of DePuy Synthes Cortex Screws (2.7 mm diameter; DePuy Synthes, Warsaw, IN, USA) and DePuy Synthes Straight and L-Fusion Plates (Locking Compression Plate 2.4/2.7 mm, lengths ranging from 37 to 62 mm; DePuy Synthes, Warsaw, IN, USA), all part of the Variable Angle LCP™ Forefoot–Midfoot System (DePuy Synthes, Warsaw, IN, USA).

### 2.4. Postoperative Rehabilitation Protocol

The postoperative protocol included reduced weight-bearing on the operated side for 30 days, followed by radiographic evaluation and a subsequent gradual increase in weight-bearing. Nonsteroidal anti-inflammatory drugs (NSAIDs) were administered postoperatively as prophylaxis against heterotopic ossification, monitoring liver and kidney function. Low-molecular-weight heparin (LMWH) was administered for approximately 5 weeks.

## 3. Results

Among 1105 DAA procedures (THA and PHA) performed during the study period, intraoperative periprosthetic proximal femoral fractures occurred in 10 cases, representing an incidence of 0.9%. All fractures included in this series occurred during the DAA; no intraoperative PPFFs were recorded with other approaches during the same period.

**Figure 1:**
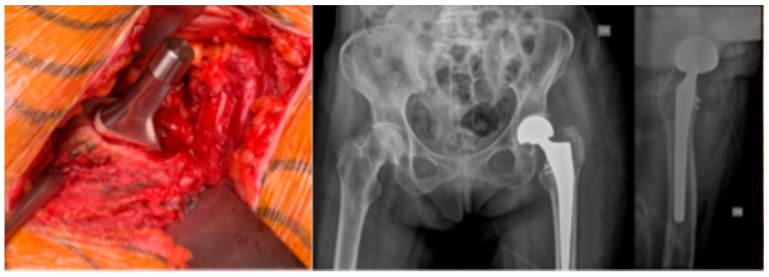
Intraoperative image showing an intraoperative periprosthetic proximal femoral fracture at the calcar level, fixed with two 2.7 mm screws, along with its postoperative X-rays at 6 months.

**Figure 2:**
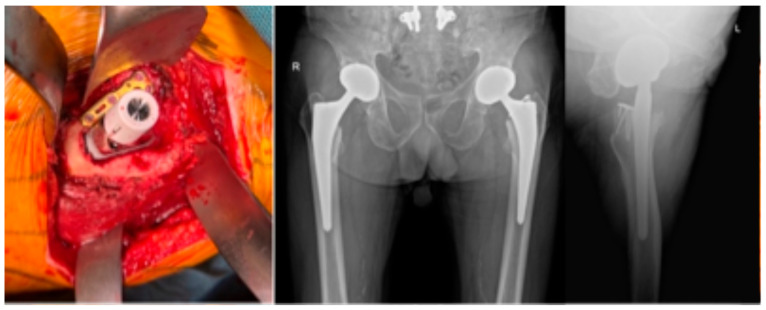
Intraoperative image showing a posterior intraoperative periprosthetic proximal femoral fracture at the base of the greater trochanter, fixed with a 37 mm L-Fusion Plate (DePuy Synthes, Warsaw, IN, USA) and four locking screws, along with its postoperative X-rays at 1 year.

**Figure 3:**
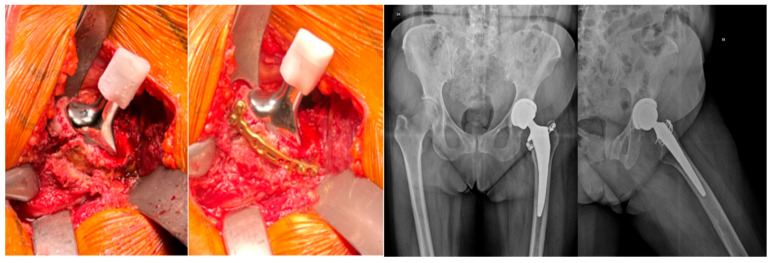
Intraoperative image showing an intraoperative periprosthetic proximal femoral fracture at the anterior cortex, before and after fixation with a 62 mm L-Fusion Plate (DePuy Synthes, Warsaw, IN, USA) and four cortex screws, along with its postoperative X-rays at 1 year.

The mean age of the patients was 72.7 years (SD 14), and the mean body mass index (BMI) was 25.2 (SD 3.3); the male-to-female ratio was 3:7. Five patients were treated using screws alone (Figure 1), while the remaining five underwent fixation with screws and plates (Figure 2 and Figure 3) (Table 2).

**Figure 4:**
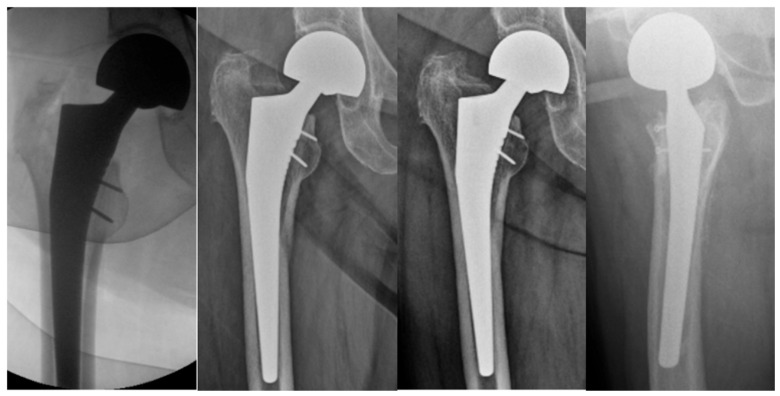
Intraoperative image showing intraoperative fluoroscopy of a periprosthetic proximal femoral fracture at the calcar level, fixed with two 2.7 mm screws, along with 2- and 5-month (AP and axial view) postoperative x-rays showing subsidence and then healing of the fracture with 1 cm of shortening.

At the final follow-up of 26 months (SD ± 11; range 8–44 months), all fractures healed. Radiographs confirmed stable implant positioning in all patients, with no cases of hardware failure. Only one patient, a 79-year-old woman unable to follow post-op indications, with a proximal femur fracture treated with a PHA, exhibited a mild fracture displacement with stem subsidence of 1 cm, which did not require revision. In this case, two calcar screws were used, and a distal fracture propagation was seen at control x-rays, probably due to an inaccurate intraoperative diagnosis and consequent fixation. The fracture entirely healed with a mild displacement (Figure 4). All five cases treated with a screw-and-plate construct, the more complex cases, healed uneventfully.

Functional outcomes improved significantly across the cohort. Harris Hip Scores (HHSs) increased from a mean of 35.4 preoperatively (SD 10.2) to 85.6 postoperatively (SD 8.3). Patients reported reduced pain and improved mobility, with no significant differences in functional recovery between fixation methods. Notably, the group treated with screws alone showed slightly higher postoperative pain, compared to the screws-and-plates group.

Complications were minimal, with one superficial wound infection that resolved with antibiotics. No instances of dislocation, implant loosening, or revision surgery were observed during the follow-up period.

Given the small sample size of five patients per subgroup, the following comparisons are purely exploratory and should not be interpreted as inferential statistics.

Subgroup analysis revealed no statistically significant differences in surgical time between the groups (screw group 133 min ± 46.8; screw-and-plate group 126 min ± 8.2; *p* = 0.62). BMI was similar across both groups (*p* = 0.82). However, patients in the screw-and-plate group were significantly older than those in the screw-only group (*p* = 0.026). Subsidence was rare and could not be reliably compared between groups due to insufficient data (Table 3).

## 4. Discussion

This study is, to our knowledge, the first to report clinical and radiological outcomes of intraoperative Vancouver A2 periprosthetic proximal femoral fractures treated during a direct anterior approach using 2.7 mm lag screws, either alone or in combination with small locking plates. All fractures in this series healed, and only one patient experienced stem subsidence, accompanied by limb shortening of 1 cm, which did not require revision surgery. In the remaining nine patients, both clinical and radiological outcomes were favorable, suggesting that this technique may represent a feasible, minimally invasive option in selected cases, though these findings should be interpreted with caution given the absence of a comparator group [2,4,5,7,21].

The incidence of calcar fracture reported by Lindahl et al. [22] and by Miettinen SS et al. [10] was 3.7%, while Abdel et al. [8], analyzing 32644 primary total hip arthroplasties, reported 564 intraoperative femoral fractures (1.7%) that occurred during placement of the femoral component (60%) and involved the calcar area in 69% of the cases.

As is known, the incidence of periprosthetic proximal femoral fractures (PPFFs) is notably higher when a direct anterior approach (DAA) is utilized [23,24] due to challenges in femoral exposure and the trajectory required for broaching the femoral canal [4]. DAA can be extended proximally and distally to improve access for fracture management, allowing for effective treatment of periprosthetic proximal femoral fractures without necessitating a change in surgical approach [4], but at the price of greater invasiveness. Few studies are available in the literature regarding periprosthetic proximal femoral fractures (PPFFs) occurring during total hip arthroplasty using the direct anterior approach (DAA). In the Hartford study [5], all intraoperative calcar fractures were treated with cerclage wiring, resulting in successful healing in most cases. According to Wilson E. J. et al. [25], cerclage cabling was the primary management strategy in 77% of intraoperative calcar fractures (IOCFs) that occurred during a DAA THA. In addition, an intraoperative stem design switch, combined with cerclage cabling, was required in 6.5% of cases to achieve adequate fixation and stability, as the original stem did not provide sufficient support.

Ineffective fixation or an undetected fracture can result in displacement, pain, nonunion, and the necessity of subsequent fixation or even a revision [26].

In addition to the widely recognized Vancouver classification system [14,27], Capello et al. proposed a modification in 2014 to highlight the significance of calcar fractures, specifically [12]. They introduced the A1 and A2 subtypes, referred to as “clamshell fractures”. This update allows for more precise differentiation between fractures involving the lesser trochanter and those affecting the calcar or medial cortex. The latter poses a greater risk to the stability of the femoral stem, making this distinction critical for appropriate treatment.

There is compelling evidence supporting the use of cerclage cable fixation for intraoperative fractures occurring around implants, which have long served and still are the standard for many [18]. However, a 2021 review highlighted a lack of comparative studies aimed at identifying the most effective method for fixing these fractures [28].

In their study, Capello et al. reported an incidence of 3.7% for intraoperative fractures, with 38 fractures out of 1039 hips operated on, 25 of which were treated with cerclage wire. Notably, only one stem showed a subsidence of 5 mm, but it remained stable for 16 years. However, the study does not specify how many of the 20 cases treated with cerclage were more complex A2-type fractures [12].

In 2024, Forlenza et al. [29] published a study comparing three different treatment options for managing intraoperative calcar fractures. They noted that, until that point, no comparative studies had examined the outcomes of various techniques for treating intraoperative, nondisplaced calcar fractures. Among 50 patients who sustained such fractures, 15 were treated with retention of the primary metaphyseal-engaging implant and placement of cerclage cables; another 15 were treated with an exchange of the primary implant for a modular, tapered-fluted stem; and a third surgeon treated 20 patients with an exchange to a fully coated, diaphyseal-engaging stem. The study found no statistically significant differences in radiographic stem subsidence among patients treated with any of the three techniques at 3 months or 1 year postoperatively. However, a high incidence (20%) of emergency department evaluations and readmissions within 90 days of surgery was observed. They also reported a high rate of reoperations (12%), primarily for infections or postoperative displacement, among patients who sustained an initially nondisplaced intraoperative calcar fracture. All reoperations occurred within the first 9 weeks following the initial total hip arthroplasty (THA).

In the context of primary THA, managing an intraoperative fracture via the anterior approach presents specific challenges, particularly for the introduction of a cerclage cable passer at the proximal aspect of the femur [6,18,30]. While cerclage wires are effective in resisting tensile forces, they are not designed to withstand shear or rotational stresses. In contrast, a screw positioned at a right angle to the fracture line aligns the fragments and offers resistance against both pullout (tension) and shear forces. Furthermore, stress relaxation, commonly associated with cerclage constructs, can lead to wire slippage. Screw fixation, by comparison, avoids this issue [18].

Maintaining adequate tension, especially when using a single wire, can be difficult. This has led some experts to recommend using two wires to ensure proper fixation [3,31]. This means a more extensive dissection and even greater difficulties.

The screw fixation method described by Johner et al. [32] demonstrated strong resistance to axial tension, allowing for early full weight-bearing. Additionally, Gauthier et al. [18] showed that interfragmentary screw fixation can achieve outcomes comparable to those of traditional cerclage systems. Graham et al. [33] and Lewis et al. [34] found that screw fixation provides even greater biomechanical stability than cables, while also causing less stripping of the periosteum and soft tissues in the proximal femur.

In certain types of proximal femoral fractures, the orientation of the fracture line may preclude the insertion of a screw perpendicular to the fracture. Based on our experience, these fractures also pose a heightened risk of detachment of the greater trochanter. This has prompted us to explore the potential benefits of using small plates and screws as a treatment option.

When a fracture is identified, one challenge that remains particularly difficult is accurately determining the true extent of the fracture line and its potential for distal propagation. In some cases, extensive dissection may be necessary to visualize the fracture line fully, or the surgeon may use fluoroscopy for assistance. Unfortunately, PPFFs in this setting are often undisplaced or minimally displaced, making fracture extension difficult to detect, especially with fluoroscopic radiological imaging.

Our elderly patient who experienced stem subsidence likely falls into this category; there was probably a distal propagation of the fracture that went unnoticed, both visually and on fluoroscopy. This situation might have warranted a different strategy. Would the use of a small plate have prevented the stem from subsiding? Alternatively, would a diaphyseal-anchored stem have been a more effective solution?

Indeed, a 2023 study by Kastner et al. [35] conducted a biomechanical investigation comparing the stability of a diaphyseal-anchored stem in the presence of an unfixed medial wall defect at the proximal femur. The craniocaudal dimension of the fracture model was aligned with the stem size. It involved 40% of the medial anchorage distance of the stem, encompassing the entire lesser trochanter along with a portion of the surrounding medial cortical wall. The researchers concluded that this type of fracture significantly decreases the axial stability of a diaphyseal-anchored stem; however, implant loosening occurred only beyond physiological stress levels, indicating that, in clinical practice, a defect of the size investigated does not pose a significant risk to the stability of diaphyseal-anchored stems.

The rationale for our choice to use plates is both straightforward and unexplored. When the fracture extends toward the posterior, posterolateral, anterior, or anterolateral regions, based on our surgical experience, we consider it to pose a greater risk than a simple calcar fracture, although this judgment has not been formally validated in this study. Another reason for opting for a plate, rather than cerclage or only screws, is poor bone quality. DePuy Synthes Straight and L-Fusion Plates are locking compression plates that provide a secure hold, even in osteoporotic bone.

It must be acknowledged, however, that the present series cannot determine whether screw-and-plate fixation is superior, equivalent, or only selectively preferable to cerclage wiring or stem revision, given the absence of a control group.

The strength of this study lies in the novelty of the use of screws and, particularly, plates for fixation of A2 intraoperative proximal femoral fractures during a direct anterior approach (DAA). This technique makes fixation much easier. The idea was born by chance and was then developed over time.

Our method and this research are subject to several limitations. First, the small sample size and retrospective, single-center design limit the statistical power and generalizability of our findings. Second, treatment allocation was not randomized; the choice between screws alone and screws plus plate was made intraoperatively based on fracture morphology and bone quality, which introduces potential selection bias. Third, different complex A2 fracture patterns were analyzed together, and the follow-up duration, although sufficient to confirm fracture healing and early stem stability, remains relatively short. Fourth, the absence of a control group treated with cerclage or stem revision prevents direct comparisons between fixation strategies. Fifth, radiographic and clinical outcome assessments were not performed by an independent observer, introducing potential observer bias. Sixth, beyond the intraoperative decision pathway described, no standardized, pre-established criteria governed the choice between screws alone and screws plus plate; this decision remained at the discretion of the operating surgeon. These limitations indicate that our results should be interpreted as preliminary and hypothesis-generating. Nevertheless, the direct anterior approach is spreading rapidly, and this method may help surgeons to get out of a problematic situation with minimal effort and invasiveness. Of course, it is mandatory to have the small, proper hardware, which is unusual for a THR procedure, available.

Our positive results, despite several limitations, encourage us to continue and expand this method to conduct more extensive and controlled studies, thereby confirming our findings and gaining more reliable evidence.

## 5. Conclusions

Intraoperative proximal femoral fractures during the direct anterior approach (DAA) pose a significant challenge, even for experienced surgeons. The use of 2.7 mm lag screws and small LCPs demonstrated favorable outcomes. This technique appears feasible and may represent a promising minimally invasive option for selected intraoperative Vancouver A2 PPFFs during DAA, with favorable short-term outcomes in our preliminary study. However, more cases, the spreading of the method, and longer follow-up are needed to confirm its effectiveness and to define the precise indications more accurately.

## Figures and Tables

**Table 1:** Patients’ demographic and preoperative characteristics.

N. Patients	10
M/F	3:7
Mean age at surgery	72.7 years (SD 14)
BMI	25.22 (SD 3.26)
Follow-up	26 months (SD 8–44)
Preoperative diagnosis	Primary osteoarthritis (5) Intracapsular femoral neck fracture (3) Secondary osteoarthritis (2)
Noble’s Flare Index classification	Champagne flute femoral canal (5) Stovepipe femoral canal (1) Normal femoral canal (4)

**Table 2:** Surgical procedure and treatment choice of intraoperative DAA-PPFF.

Hip replacement	THA (7) HA (3)
Femoral implant type	Polarstem uncemented (4) Polarstem cemented (2) Actis uncemented (2) Corail uncemented (2)
Acetabular implant type	Trident cup (7)
Fixation method	Screw 2.7 mm (5) L-Fusion Plate and Screw 2.7 mm (5)

**Table 3:** Patients’ outcomes. †Preoperative HHS not available for patients presenting with acute femoral neck fracture.

ID	Group	HHS Preop	HHS Post-Op (6 Months)	VAS (6 Months)	Subsidence	Complications	Operative Time
**1**	Screw	21.7	95	2	No	No	101
**2**	Screw	26	70.6	2	No	No	82
**3**	Screw	29.8	75.8	1	No	No	199
**4**	Screw	N/A	89.1	3	Yes	Subsidence	160
**5**	Screw	N/A	80.2	2	No	No	121
**6**	Plate + Screw	48.7	92	1	No	No	115
**7**	Plate + Screw	40.6	83.2	2	No	Wound infection	136
**8**	Plate + Screw	N/A	86.1	2	No	No	124
**9**	Plate + Screw	35.2	87.6	1	No	No	131
**10**	Plate + Screw	48.7	96.5	1	No	No	121

## Data Availability

The data that support the findings of this study are available from the corresponding author upon reasonable request. Due to privacy and ethical restrictions, individual patient data are not publicly available.

## Abbreviations

The following abbreviations are used in this manuscript:

PPFF	Periprosthetic Proximal Femoral Fracture
THA	Total Hip Arthroplasty
DAA	Direct Anterior Approach
PHA	Partial Hip Arthroplasty
HHS	Harris Hip Score
BMI	Body Mass Index
GT	Greater Trochanter
SD	Standard Deviation
NSAIDs	Nonsteroidal Anti-Inflammatory Drugs
LMWH	Low-Molecular-Weight Heparin
FU	Follow-Up
